# Single-cell and immune-context integration identifies basement-membrane/metastasis signatures that sharpen bladder-cancer diagnosis and prognosis

**DOI:** 10.1007/s12672-026-04440-3

**Published:** 2026-01-14

**Authors:** Ji Chen, Xiaobing Liu, Rongyin Ren, Renzheng Yi, Xiongfeng Zhang, Chaoqun Xie, Xinyu Liu, Weibing Long

**Affiliations:** 1 Department of Urology, Loudi City Central Hospital, Loudi, 417000 Hunan China; 2 Department of Otolaryngology Head and Neck Surgery, Loudi Central Hospital, Loudi, 417011 Hunan China

## Abstract

**Background:**

Bladder cancer(BLCA) has a high recurrence rate and metastasis, and its process is closely related to basement membrane remodeling. we developed an interpretable prognostic model based on metastasis–basement membrane–related genes (MBRGs) to enhance clinical and personalized treatment strategies.

**Method:**

Differentially expressed MBRGs from TCGA and GEO cohorts were analyzed. Prognostic genes were identified by univariate Cox and LASSO regression. A six-MBRG risk model was built and externally validated. SHAP analysis quantified feature contributions. Functional enrichment analyzed via GSEA and KEGG. Immune cell profiles estimated with CIBERSORT and ssGSEA. Immunotherapy response predicted using TMB, TIDE, and mutation frequency. Single-cell and spatial transcriptomics localized key genes to cancer-associated fibroblasts(CAFs).

**Results:**

Through analysis of metastasis and basement membrane-associated DEGs, 18 candidate MBRGs were identified and refined via univariate Cox and SHAP to a 6-gene signature (SERPINF1, DDR2, SLIT2, HSPG2, ECM1, RECK). This signature demonstrated prognostic power with AUCs of 0.638–0.674 in TCGA and 0.602–0.742 in GEO cohorts. A clinical nomogram achieved an AUC of 0.827. The high-risk group exhibited elevated M2 macrophages and TIDE scores(a computational metric for predicting tumor immune evasion and immunotherapy response), whereas the low-risk group showed enriched CD8⁺ T cells. Drug assays indicated dasatinib sensitivity in low-risk patients, and LGK974, LY2109761, and Wnt-C59 in high-risk patients. Single-cell RNA-seq and IHC confirmed CAF-specific overexpression of DDR2 and SERPINF1.

**Conclusion:**

The MBRG-based model effectively predicts BLCA prognosis, integrates mechanisms of basement membrane remodeling, EMT, and immune suppression, and identifies DDR2 and SERPINF1 in CAFs as potential targets for personalized therapy.

**Supplementary Information:**

The online version contains supplementary material available at 10.1007/s12672-026-04440-3.

## Introduction

BLCA represents one of the most prevalent types of urinary cancer on a global scale. According to GLOBOCAN 2022, there are approximately 614,000 new cases annually, making it the ninth most frequently diagnosed cancer globally. Over the past two decades, both incidence and mortality rates have increased significantly [1]. Epidemiological studies have indicated a notable gender disparity in BLCA, with men affected approximately three to four times more often than women. Male patients often develop the disease at a younger age, closely associated with risk factors such as smoking, occupational chemical exposure, and chronic bladder inflammation [1, 2]. More than 90% of BLCA are classified as urothelial carcinoma, characterized by significant clinical variability. Clinically, BLCA is categorized into two primary subtypes based on invasion depth: This refers to two distinct types of BLCA, namely non-muscle invasive (NMIBC) and muscle-invasive bladder cancer (MIBC) [3]. Approximately 75% of newly diagnosed BLCA are NMIBC. Although surgical removal combined with intravesical therapy achieves good local control, managing these cases remains challenging due to a high recurrence rate (> 60% within five years) and a significant annual progression rate (5–15%) [4]. MIBC accounts for approximately 25% of cases, where cancer cells invade deeper tissues beyond the bladder lining. At diagnosis, about half already have microscopic spread [5]. Despite aggressive treatment involving bladder removal and chemotherapy, 30–50% develop distant metastases, with fewer than 50% surviving beyond five years [6]. Metastatic lesions frequently occur in vital organs such as the lungs, bones, and liver, severely complicating treatment and significantly worsening patient outcomes [2].

The metastatic cascade significantly contributes to BLCA mortality and involves three critical stages: modification of the original tumor site environment, survival of cancer cells in circulation, and establishment at distant organs [7, 8]. The disruption of the basement membrane is regarded as a pivotal initial event, tightly controlled by various biological signals and mechanical factors [9–11]. Although radical surgery and systemic treatments extend survival in some patients, approximately 50% of MIBC patients ultimately experience treatment failure due to metastatic recurrence, particularly evident in advanced cases [5]. Metastasis in BLCA begins with increased tumor cell migration. Recent research highlights the role of cell membrane protrusions, driven by dynamic cytoskeletal remodeling and regulated precisely by specific molecular signals [12, 13]. On a molecular level, the activation of specific signaling pathways not only enhances cell mobility but also facilitates cancer spread by triggering cellular transitions that enable cells to migrate effectively [14]. Recent studies also suggest novel molecular interactions that simultaneously regulate the cell’s structural remodeling and degradation of surrounding tissues, shedding new light on how BLCA cells specifically metastasize [7].

Although significant advances in understanding BLCA biology have occurred, clinical management still faces challenges. Approximately 30% of patients are diagnosed at advanced stages due to nonspecific early symptoms. Additionally, modern immunotherapies, though promising, achieve an overall response rate of only about 20%, primarily because of drug resistance [5, 15]. These issues fundamentally relate to changes in the immune environment within tumors and abnormal gene regulation mechanisms [16, 17].

Reprogramming of the tumor microenvironment significantly promotes metastatic tumor establishment. Tumor-associated macrophages (TAMs) secrete chemokines such as CCL20, creating a niche conducive to metastasis. This activity has dual effects: directly activating genes that enhance tumor cell invasion and facilitating new blood and lymphatic vessel formation, thereby providing channels for cancer spread [18]. Temporal and spatial dynamics within this cell-microenvironment interaction network partly explain why single-target therapies frequently lead to drug resistance. Although immune checkpoint inhibitors (ICIs) have improved outcomes for some advanced patients, approximately 43% of PD-L1-positive patients do not respond to treatments like avelumab [19]. This resistance is likely linked to heterogeneity in the tumor immune microenvironment.

Basement membrane remodeling, a critical link between tumor invasion and immune evasion, is increasingly recognized. Matrix metalloproteinase (MMP)-mediated degradation of laminin not only promotes cancer cell invasion but also releases extracellular matrix fragments such as endostatin, stimulating new blood vessel formation [20, 21]. Additionally, agrin, a basement membrane component, activates regulatory T-cell infiltration through integrin αvβ3-MAPK signaling, fostering an immunosuppressive environment [22]. Tumor cells can physically traverse the basement membrane via elongated protrusions driven by integrins and myosin II-generated contractile forces [23]. Integrative analysis of basement membrane and metastasis provides robust efficacy in tumor assessment [24]. Currently, there is a lack of multidimensional prognostic tools in BLCA capable of simultaneously evaluating basement membrane-related gene expression patterns and metastatic potential, which limits the development of precision therapeutic strategies.

Thus, this study innovatively develops a prognostic model based on metastasis and basement membrane-related genes (MBRGs). We hypothesize that the expression profile of MBRGs reflects metastatic potential and the dynamic state of the tumor immune microenvironment. By integrating multi-omics data, we aim to evaluate the prognostic significance, immune regulation capacity, and predictive therapeutic response of MBRGs, thereby offering a new decision-making framework for precision medicine in BLCA.

## Materials and methods

### Data resource

A total of 415 BLCA patients were selected from The Cancer Genome Atlas (TCGA), comprising 396 tumor and 19 normal samples. Expression data, clinical information, and mutation data were retrieved for analysis. An external validation dataset, GSE32894, consisting of 308 BLCA samples, was downloaded from the Gene Expression Omnibus (GEO). The gene expression data were generated using the Illumina HumanHT-12 V3.0 expression beadchip platform (GPL6947). Basement membrane-related genes were obtained from previously published research [25]. Metastasis-related genes with scores above 10 were selected from the Genecards database.

### Differential expression analysis and gene selection in TCGA-BLCA

We retrieved FPKM expression profiles for 415 BLCA samples (396 tumors and 19 normals) from TCGA, encompassing 59,427 genes. The determination of differential expression was achieved by employing the limma R package, which utilised thresholds of log₂FC ≥ 1 and FDR < 0.05. This process resulted in the identification of 2,615 DEGs [26]. From GeneCards, 1,907 metastasis-related genes were obtained, and 224 basement membrane (BM) genes were sourced from prior literature [25]. The intersection of 2,615 DEGs, 1,907 metastasis-related genes, and 224 basement membrane genes yielded 18 overlapping candidates. Subsequently, univariate Cox regression analysis (*p* < 0.05) was performed. This analysis identified 12 DEGs significantly associated with metastasis and basement membrane–related prognosis [27].

### Model construction and SHAP interpretation

The construction of a multivariate Cox proportional hazards model incorporating 12 metastasis-basement membrane-related genes (MBRGs) was achieved by utilising the training cohort [28]. Bidirectional stepwise regression identified the optimal gene panel and estimated coefficients (β₁, …, βₚ). After model construction, SHAP analysis was applied to interpret model predictions. This approach quantified each gene’s contribution to individual risk scores and visualized feature importance. Regression coefficients, hazard ratios (with 95% confidence intervals), and p-values were extracted from the model summary to assess statistical significance and prognostic relevance. Based on SHAP-derived baseline values, samples were stratified into high- and low-risk groups. A dataset including survival time, survival status, risk score, and risk group assignment was generated for subsequent survival and ROC analyses.

### SHAP method

We employed SHapley Additive exPlanations (SHAP), a post-hoc interpretability technique that balances global and local explanations by computing each feature’s marginal contribution to the model output. Rooted in game theory and Shapley values [29], SHAP treats features as “players” contributing additively to predictions. By calculating SHAP values for each feature, we quantified their impact on overall model outputs and revealed the internal relationships between features and predictions at both the cohort and individual sample levels. This method is conceptually aligned with the kernel-based approach (KernelSHAP).

### Nomogram construction and independent prognostic analysis

The identification of independent prognostic factors in BLCA was achieved by implementing univariate and multivariate Cox regression analyses. Based on multivariate results, a nomogram was generated with the RMS package to aid clinical decision-making. The nomogram integrated age, grade, TNM stage, and risk score from the TCGA-BLCA dataset. Calibration curves at 1, 3, and 5 years assessed predictive performance [30], and the concordance index validated model accuracy.

### Functional and pathway enrichment analysis

The process of conducting GO and KEGG enrichment analyses was performed on high- and low-risk BLCA cohorts, utilising the clusterProfiler method [31]. Subsequently, Gene Set Enrichment Analysis (GSEA) was conducted using the MSigDB c2.cp.kegg.v2022.1.Hs.symbols.gmt collection. This analysis identified pathways showing consistent but non-significant differential expression trends. These results were used to explore the association between prognostic gene expression and the epithelial–mesenchymal transition (EMT) pathway. The EMT pathway has been demonstrated to promote basement membrane invasion and metastatic potential [32].

### Immune profiling and immunotherapy

The quantification of the abundance of 22 immune cell types was conducted in each BLCA sample using ssGSEA via the GSVA package. Furthermore, we implemented the CIBERSORT [33] and ssGSEA [34] methodologies to comprehensively assess the immune microenvironment in high- versus low-risk groups. The expression levels of immune checkpoint genes were then compared between the groups in order to predict how sensitive they would be to the therapeutic treatment.

### Mutation analysis

Somatic mutation and CNV data for all patients were extracted from TCGA. Focusing on key prognostic genes, we calculated mutation frequencies and CNV distributions. Patients were stratified into high- and low-risk groups by Cox model risk scores to facilitate a comparison of genetic alteration profiles. Tumor mutational burden (TMB) was calculated for each patient. Based on the median TMB value, patients were classified into high- and low-TMB groups. Combining TMB status with risk stratification resulted in four subgroups (high/low risk × high/low TMB). Kaplan–Meier survival curves and log-rank tests were utilised to evaluate overall survival differences, thereby elucidating how risk stratification and mutation burden jointly influence prognosis.

### Drug sensitivity analysis

Using gene expression profiles from BLCA samples, we employed the pRRophetic algorithm in conjunction with the Genomics of Drug Sensitivity in Cancer (GDSC) database to estimate the relative drug response (predicted IC₅₀ values) for multiple anticancer agents. Patients were stratified into high- and low-risk groups according to the MBRG risk score. Predicted IC₅₀ values were derived by mapping tumor transcriptomic features to drug response models trained on cancer cell line data, enabling a comparative assessment of potential chemotherapy and targeted therapy sensitivity between risk groups. In addition, drug target genes were retrieved from DrugBank, and their expression patterns were analyzed across risk groups to provide biological context for the predicted drug response differences. Prior to drug sensitivity prediction, gene expression data were log2-transformed and normalized to ensure comparability across samples. To account for potential batch effects arising from different data sources and platforms, batch effect correction was performed using the ComBat algorithm (sva package), with dataset origin specified as the batch variable. The batch-corrected expression matrix was subsequently used as input for the pRRophetic algorithm, which estimates relative drug sensitivity by mapping tumor transcriptomic profiles to drug response models trained on GDSC cancer cell line data.

### Multiplex immunohistochemistry

Clinical specimens were obtained with approval from the Clinical Trial Ethics Committee of Loudi Central Hospital (Ethics ID: 2021-Ethics Review [Scientific Research]-039). Informed consent was obtained from all participants. For participants under the age of 18, informed consent was obtained from their parents and/or legal guardians. Multiplex immunohistochemistry (mIHC) was performed using the Opal four-color manual IHC kit (PerkinElmer, NEL810001KT) following the manufacturer’s protocol. The primary antibodies used were DDR2 (Proteintech, 67126-1-Ig), SERPINF1 (Proteintech, 26045-1-AP), CD68 (Abcam, ab213363), and α-SMA (Abcam, ab124964). Multispectral images were acquired using a Vectra imaging system and analyzed with inForm software (version 2.4.8).

### Single-cell RNA sequencing and spatial transcriptome analysis

We performed scRNA-seq analysis of DDR2 and SERPINF1 in BLCA using the GSE129845 dataset from GEO [35], which comprises 12,423 cells from three samples.(accession number GSE285715). Analyses were conducted in R using the Seurat package. Raw UMI counts were normalized and scaled using the SCTransform function. Highly variable genes were identified during this process. Dimensionality reduction was performed using RunPCA, and unsupervised clustering was carried out with FindNeighbors and FindClusters based on the first 30 principal components (default parameters).

### Software and statistical analysis

All statistical analyses were performed using R software (version 4.2.2). Differential expression analysis was conducted using the limma package (version 3.52.4). Survival analyses were performed with the survival (version 3.4-0) and rms (version 6.6-0) packages. Functional enrichment analyses were carried out using clusterProfiler (version 4.12.6). Immune infiltration analyses were performed using GSVA (version 1.42.0) and CIBERSORT. Single-cell RNA-seq analyses were performed using Seurat (version 4.3.0).Multiple testing correction was applied where appropriate to control the false discovery rate (FDR). Unless otherwise specified, p-values were adjusted using the Benjamini–Hochberg (BH) method. Adjusted p-values (or FDR q-values) < 0.05 were considered statistically considered significant.

## Results

### Prognostic gene identification and analysis

**Fig. 1:**
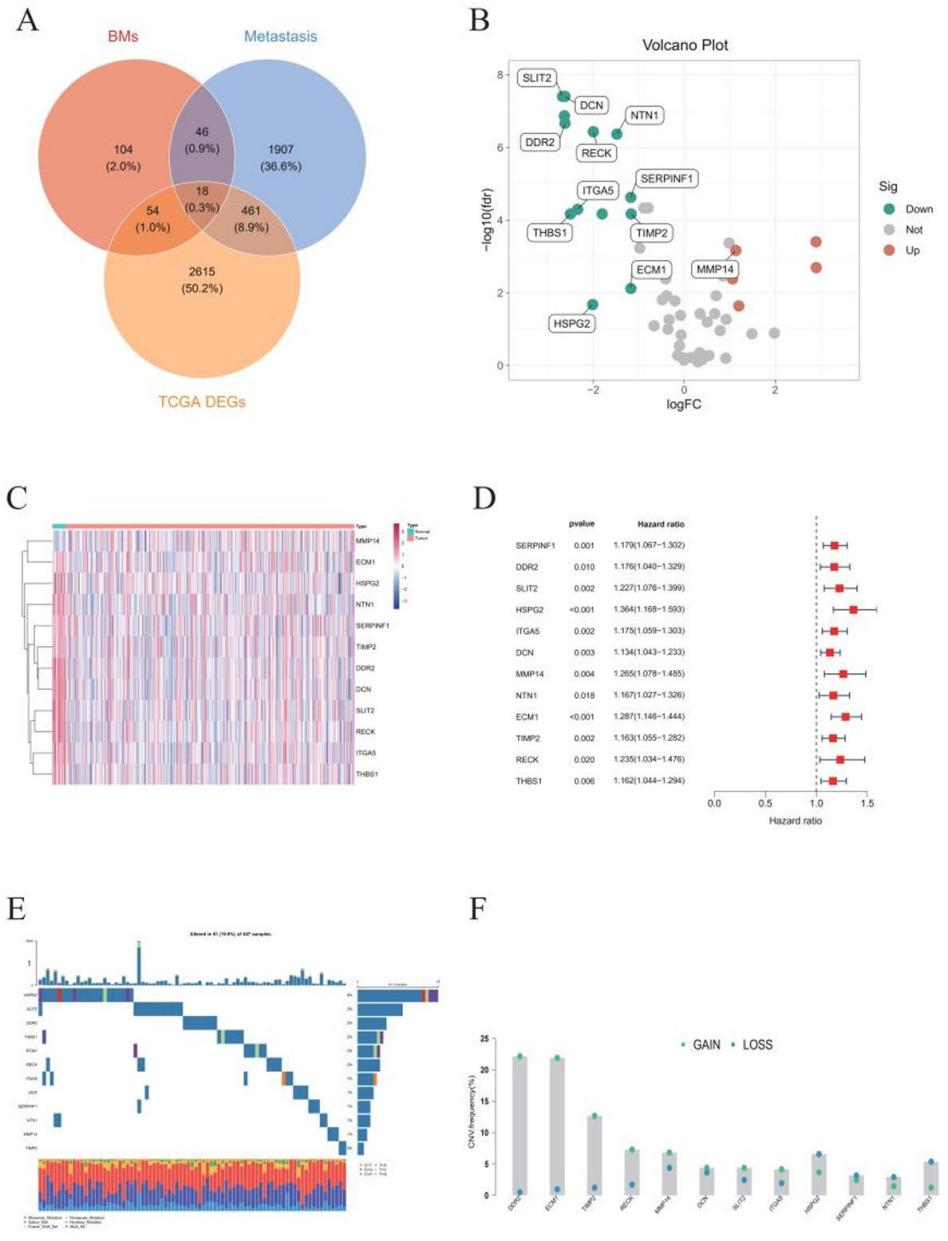
Prognostic and genomic characterization of metastasis- and basement membrane-related differentially expressed genes in BLCA. **A** Venn diagram depicting intersections among differentially expressed genes, metastasis-related genes, and basement membrane genes. **B** Volcano plot illustrating differential expression of prognostic genes associated with metastasis and basement membrane in tumor versus normal tissues. **C** Comparative expression of 12 selected metastasis- and basement membrane-related prognostic genes between tumor and normal tissues. **D** Forest plot showing the prognostic significance of the 12 genes. **E** Mutation landscape analysis of these 12 prognostic genes using MAF tools. **F** Copy number variation (CNV) analysis of the 12 prognostic genes associated with metastasis and basement membrane remodeling

We analyzed expression data for 426 BLCA patients from TCGA (407 tumors, 19 normals), covering 59,427 genes (FPKM). Differential expression analysis identified 2,615 DEGs. From GeneCards, we retrieved 1,907 metastasis-related genes and 224 basement membrane (BM) genes. Intersecting these with the DEGs yielded 18 overlapping candidates (Fig. 1A), of which five were upregulated and 13 downregulated (Fig. 1B). Univariate Cox regression identified 12 prognostic genes significantly associated with overall survival (HR > 1, *p* < 0.05). Eleven were downregulated in tumors (Fig. 1C, D), suggesting that low expression predicts poor prognosis. Mutation and CNV analysis in 407 patients showed alterations in these genes in 81 cases (19.9%), including HSPG2 (6%), SLIT2 (3%), DDR2 (2%), THBS1 (2%), ECM1 (2%), RECK (2%), ITGA5 (1%), DCN (1%), SERPINF1 (1%), NTN1 (1%), and MMP14 (1%) (Fig. 1E). DDR2, ECM1, TIMP2, and RECK exhibited predominantly amplifications, whereas HSPG2 and THBS1 were mainly deleted (Fig. 1F). Survival analysis confirmed that higher expression of these prognostic genes correlates with worse survival (Fig. S1).

### Model construction and SHAP analysis

We selected six MBRGs (SERPINF1, DDR2, SLIT2, HSPG2, ECM1, RECK) from 12 prognostic genes identified by univariate Cox regression to build a multivariate Cox model. Risk coefficients are listed in Table S1, and the risk score was calculated as:

$$ \begin{aligned} {\mathrm{Risk}}{\mkern 1mu} {\mathrm{score}}{\mkern 1mu} & {\text{ = }}{\mkern 1mu} \left( {{\mathrm{0}}{\mathrm{.258*SERPINF1exp}}{\mathrm{.}}} \right) - \left( {{\mathrm{0}}{\mathrm{.522*DDR2exp}}{\mathrm{.}}} \right) \\ {\mkern 1mu} & {\text{ + }}{\mkern 1mu} \left( {{\mathrm{0}}{\mathrm{.305*SLIT2exp}}{\mathrm{.}}} \right){\mkern 1mu} {\text{ + }}{\mkern 1mu} \left( {{\mathrm{0}}{\mathrm{.404*HSPG2exp}}{\mathrm{.}}} \right){\mkern 1mu} \\ & {\text{ + }}{\mkern 1mu} \left( {{\mathrm{0}}{\mathrm{.288*ECM1exp}}{\mathrm{.}}} \right) - \left( {{\mathrm{0}}{\mathrm{.269*RECKexp}}{\mathrm{.}}} \right) \\ \end{aligned} $$

**Fig. 2:**
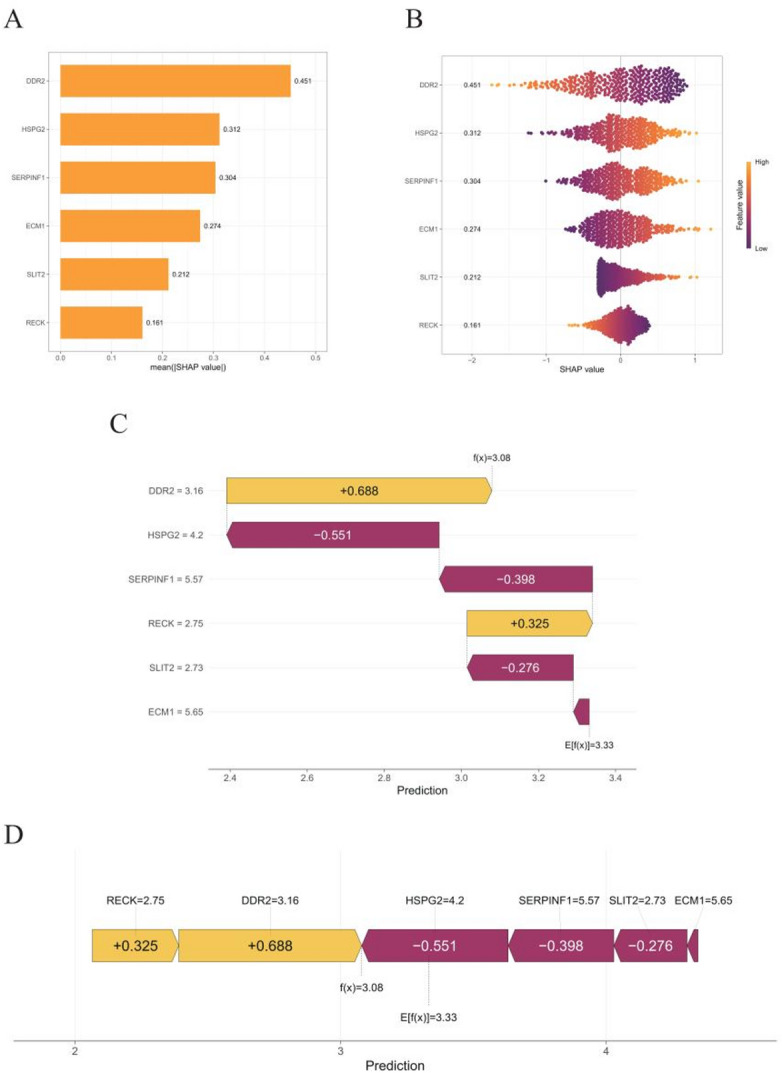
SHAP analysis of gene contributions and personalized risk assessment in BLCA. **A** Bar plot ranking gene importance based on SHAP values calculated by the SHAP interpretation model. **B** SHAP bee-swarm plot depicting gene influences on model-derived risk scores. **C**, **D** Waterfall and force plots illustrating personalized predictions and interactions among genes impacting individual risk assessments

We applied SHapley Additive exPlanations (SHAP) to interpret this model. Each gene’s contribution was quantified by its mean absolute SHAP value and ranked: DDR2 (0.451) had the greatest impact, followed by HSPG2 (0.312), SERPINF1 (0.304), ECM1 (0.274), SLIT2 (0.212), and RECK (0.161) (Fig. 2A). The SHAP summary plot (Fig. 2B) shows that high DDR2 and RECK expression decreases the risk score (negative SHAP), whereas elevated HSPG2, SERPINF1, ECM1, and SLIT2 increase it (positive SHAP). Local interpretations demonstrate personalized predictions: for one representative sample, the baseline risk was 3.33; positive contributions from DDR2 (+ 0.688) and RECK (+ 0.325) raised the score, while negative contributions from HSPG2 (–0.551), SERPINF1 (–0.398), and SLIT2 (–0.276) offset the increase, resulting in a final score of 3.08 and classifying the patient as low risk (Fig. 2C). The force plot (Fig. 2D) visualizes how these genes collectively influence the individual risk assessment.

### Validation of the prognostic model

**Fig. 3:**
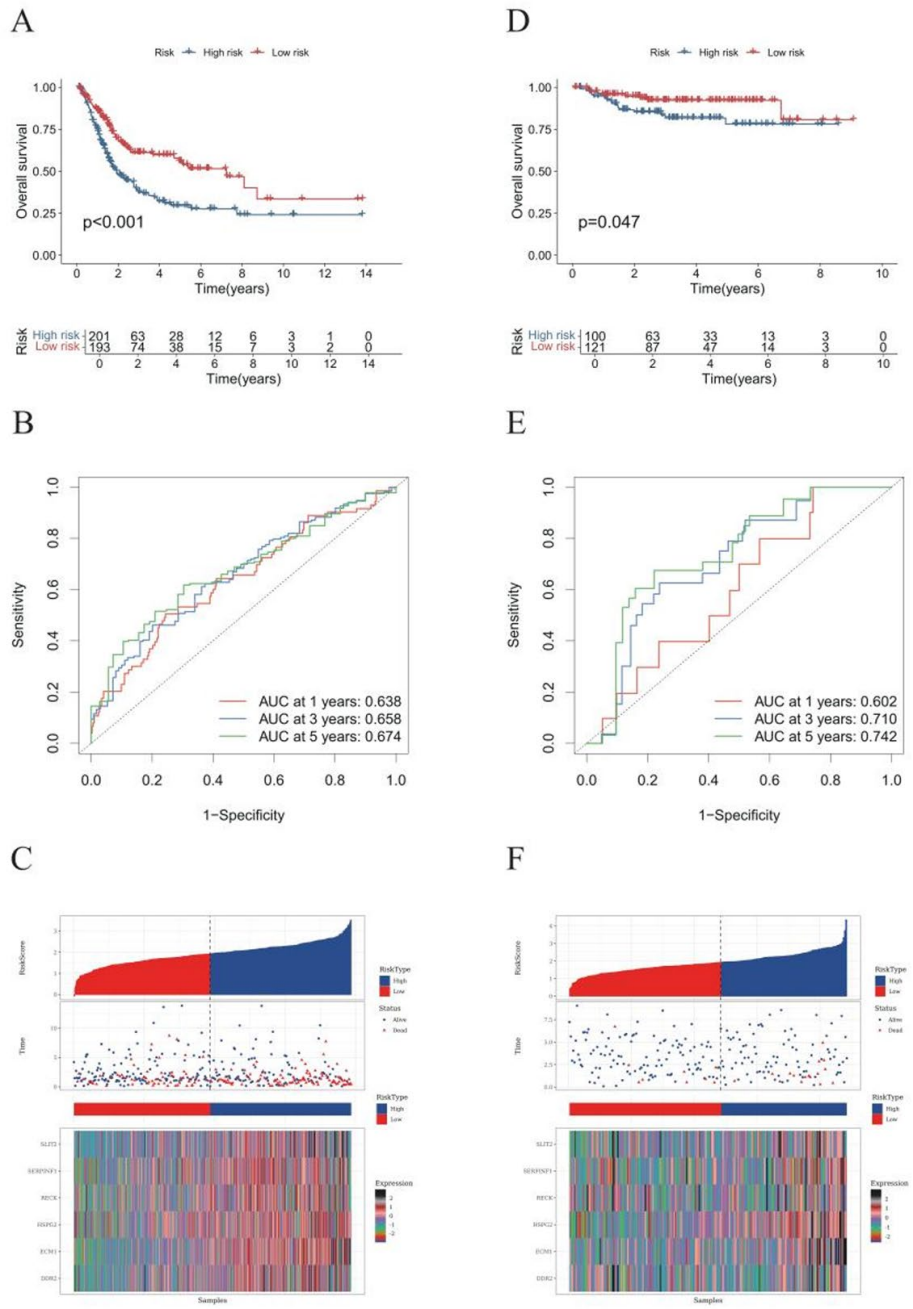
Construction and validation of the metastasis- and basement membrane-related gene prognostic model. **A** Kaplan-Meier survival curves comparing overall survival (OS) between high- and low-risk groups in TCGA dataset. Patients in the high-risk group had significantly worse OS compared to those in the low-risk group (hazard ratio [HR] = 3.45, 95% confidence interval [CI]: 2.50–4.76; log-rank *p* < 0.001). **B** Combined time-dependent ROC curves of the MBRG risk model in the TCGA cohort. The 1-, 3-, and 5-year ROC curves are overlaid in a single plot to enable direct visual comparison of predictive performance over time. **C** Association analysis between risk scores and survival status in TCGA dataset. **D** Kaplan-Meier survival curves for OS comparison between high- and low-risk groups in GEO dataset. **E** Combined time-dependent ROC curves of the MBRG risk model in the GEO validation cohort. The overlaid 1-, 3-, and 5-year ROC curves demonstrate consistent and robust discrimination across different follow-up periods. **F** Association analysis between risk scores and survival status in GEO dataset

Kaplan–Meier survival analysis (log-rank *P* < 0.001) in the TCGA cohort revealed a pronounced overall survival advantage in the low-risk group compared to high-risk counterparts (Fig. 3A), underscoring the robust risk stratification capacity of the MBRG-derived model. The time-dependent receiver operating characteristic (ROC) analysis yielded area under the curve (AUC) values of 0.638, 0.658, and 0.674 for predicting 1-, 3-, and 5-year overall survival, respectively (Fig. 3B). These results indicate a moderate to good discriminative ability of the MBRG-derived risk model that remains stable across increasing follow-up durations, reflecting consistent predictive performance over time.

We then ranked patients by risk score and plotted score versus survival status. Higher risk scores correlated with increased mortality events, whereas low-risk patients exhibited longer survival times (Fig. 3C). PCA and t-SNE both showed clear separation between high- and low-risk groups in two-dimensional space (Fig. 3D), further supporting the model’s ability to distinguish patient subgroups.

To assess generalizability, we applied the same risk-scoring formula to an independent BLCA cohort from the GEO database. Kaplan–Meier survival analysis again revealed significantly poorer overall survival in the high-risk group (*P* = 0.047; Fig. 3E). In this validation cohort, time-dependent ROC analysis produced AUC values of 0.602, 0.710, and 0.742 at 1, 3, and 5 years, respectively (Fig. 3E), demonstrating improved discriminative performance at longer follow-up times and further supporting the temporal robustness and cross-cohort generalizability of the MBRG risk model. The joint visualisation of risk score, survival status, and expression of the six prognostic genes demonstrated a positive correlation between higher scores and greater mortality risk (Fig. 3F). PCA and t-SNE further revealed distinct clustering of high- and low-risk samples (Fig. 3H), underscoring the model’s robustness and classification power.

### Clinical feature analysis and nomogram construction

**Fig. 4:**
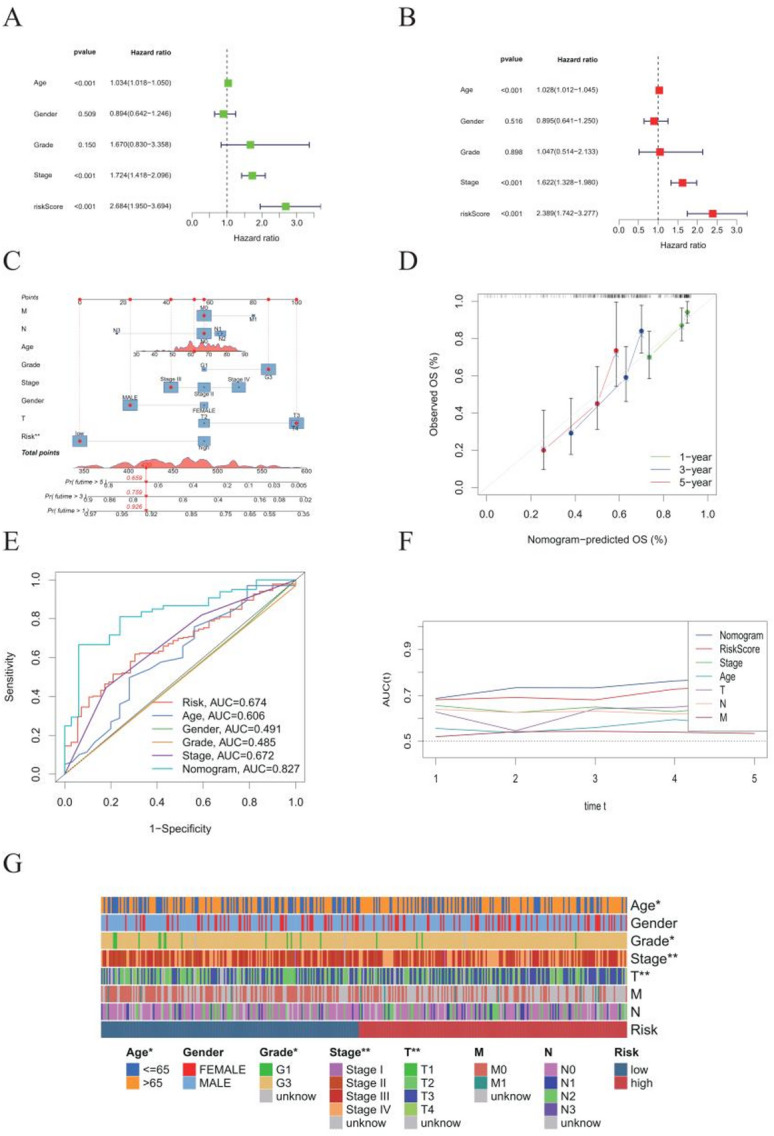
Independent prognostic analysis and predictive nomogram development based on risk scores. **A** Univariate Cox regression analysis in TCGA-BLCA cohort. **B** Multivariate Cox regression analysis in TCGA-BLCA cohort. **C** Construction of an individualized predictive nomogram integrating age, gender, tumor grade, clinical stage, TNM classification, and risk scores for predicting 1-, 3-, and 5-year OS. **D** Calibration curve analysis of the nomogram model. **E** ROC curves assessing prognostic prediction power of individual clinical factors. **F** Dynamic AUC analysis comparing predictive accuracy of the nomogram and individual factors. **G** Heatmap illustrating the distribution of age, gender, tumor grade, clinical stage, TNM classification, and risk groups in BLCA patients

In TCGA-BLCA patients, multivariate Cox regression incorporating age, sex, grade, TNM stage, and the MBRG risk score identified age > 65 years (*P* < 0.05), high grade (G3 vs. G1, *P* < 0.05), advanced clinical stage (III/IV vs. I/II, *P* < 0.01), and T3/4 vs. T1/2 (*P* < 0.01) as independent predictors of poor overall survival (Fig. 4A). Univariate and multivariate analyses were conducted to ascertain the independence of age, stage, and the MBRG-derived risk score as prognostic factors for OS. The results of these analyses revealed that all three factors were statistically significant (*P* < 0.01; Fig. 4B, C). We then built a nomogram integrating these factors to improve individualized prognostic assessment (Fig. 4D). Internal calibration curves at 1, 3, and 5 years showed excellent agreement (Fig. 4E). When combined with clinical variables, the nomogram achieved an AUC of 0.827, demonstrating high predictive accuracy and reliability (Fig. 4 F, G).

### Positive correlation between MBRG score and EMT activity

**Fig. 5:**
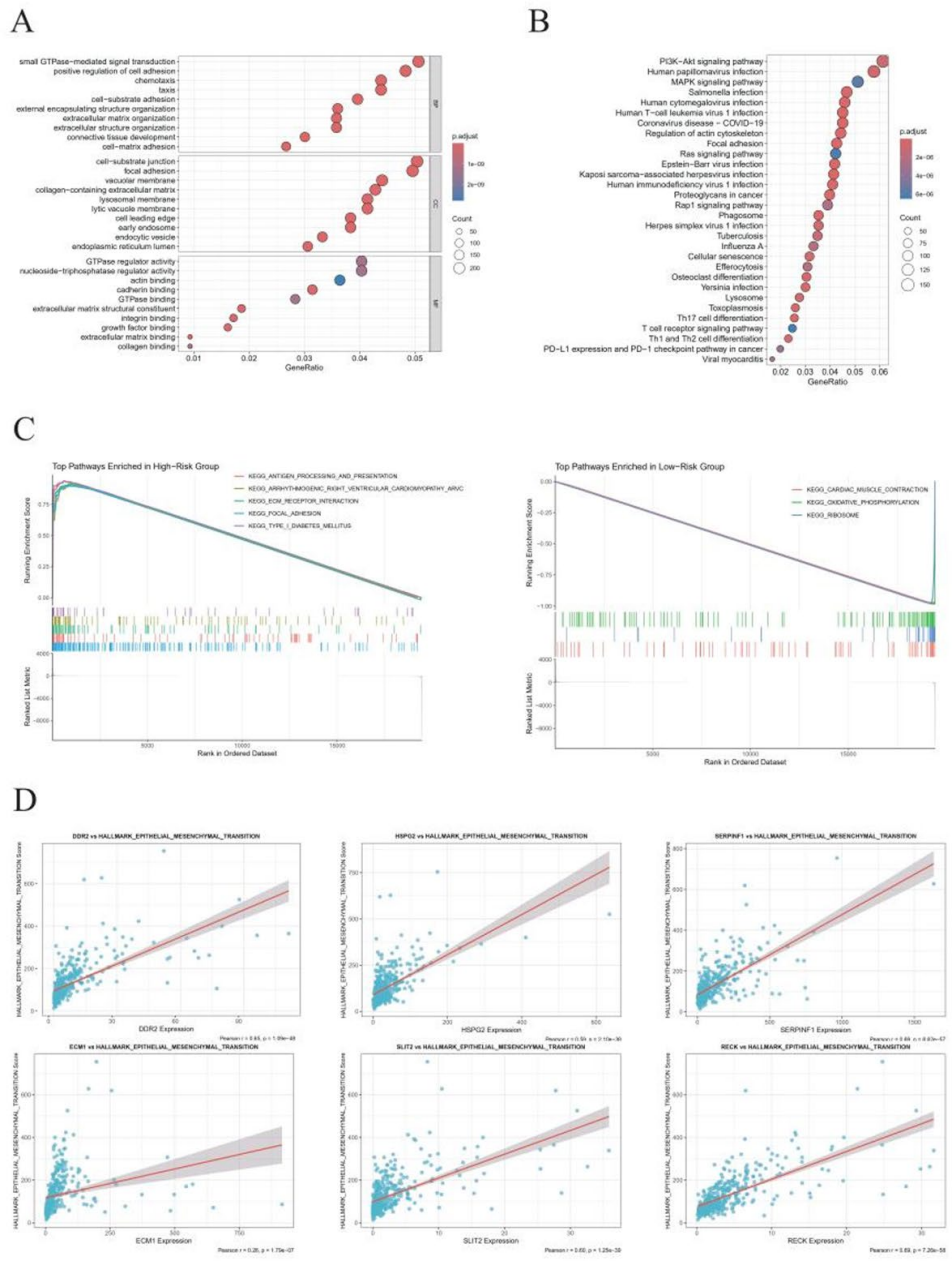
Functional enrichment analysis and correlation of metastasis- and basement membrane-related genes with EMT pathway. **A** GO enrichment analysis. **B** KEGG pathway enrichment analysis. **C** GSEA pathway enrichment analysis based on risk scores. **D** Correlation analysis between EMT pathway activation and expression of six signature genes

During the EMT, there is a loss of basement membrane integrity, which is critical for tumour invasion and metastasis. We therefore assessed the relationship between MBRG risk scores and EMT signaling. KEGG and GO enrichment analyses revealed significant overrepresentation of DEGs in pathways related to tumorigenesis and metastasis (Fig. 5A, B). GSEA showed that high-risk tumors are enriched for immune-evasion and invasion-related pathways, whereas low-risk tumors are enriched for metabolic pathways (Fig. 5C; Table S2). These findings underscore the pivotal role of BM remodeling in BLCA progression. Additionally, GSEA suggests that poor prognosis in high-risk patients is largely driven by extracellular matrix reprogramming. Finally, correlation scatterplots demonstrated strong positive associations between EMT pathway activity and expression of DDR2, SERPINF1, and RECK (Fig. 5D).

### Immunological profiling

**Fig. 6:**
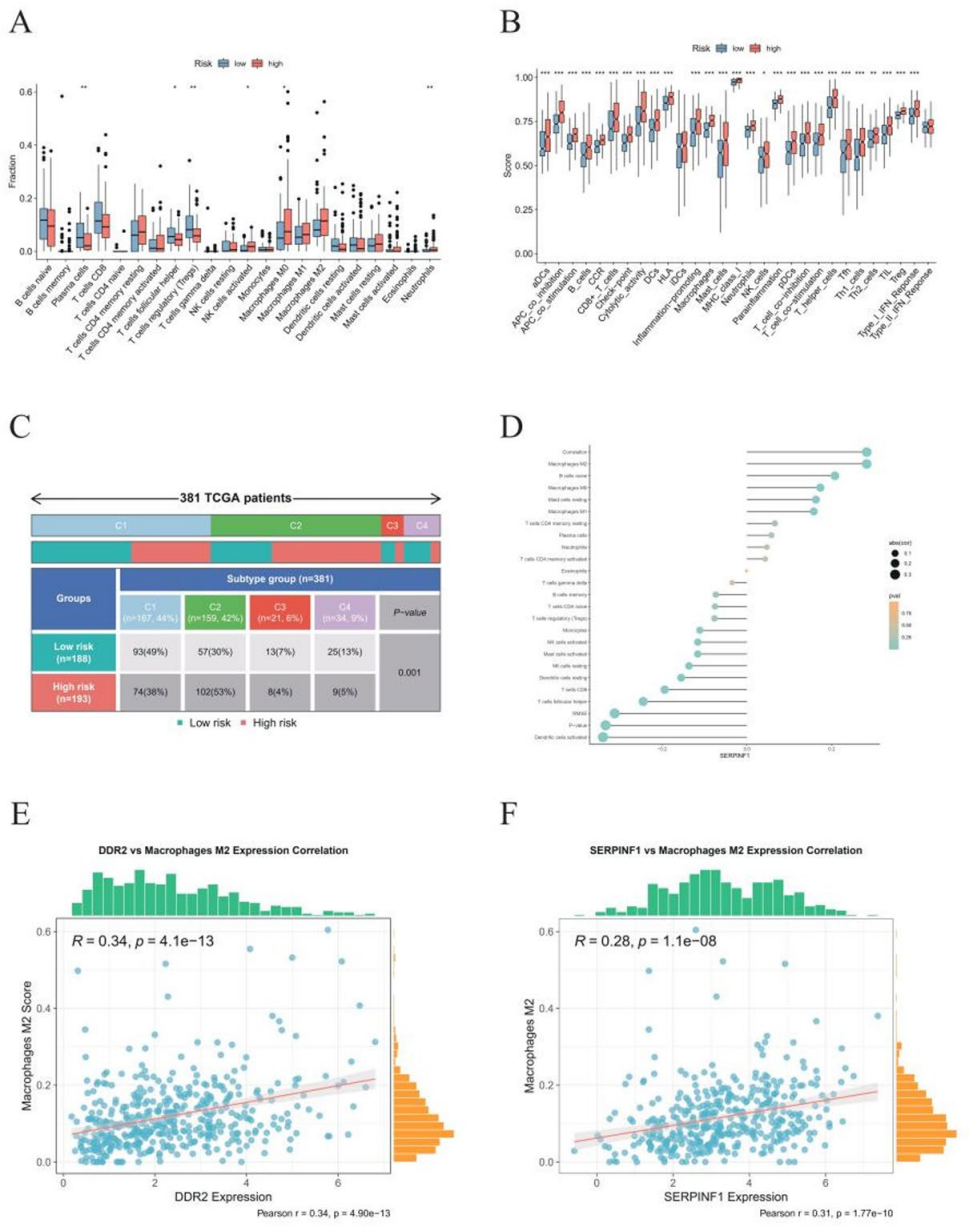
Immune characteristics of BLCA patients stratified by risk scores. **A** Differential immune cell infiltration between risk groups. **B** Comparative analysis of immune pathway activities in high- and low-risk groups. **C** Correlation analysis between risk scores and immune subtypes. **D** Associations between SERPINF1 expression and multiple immune cells. **E** Scatterplot showing correlation between SERPINF1 expression and M2 macrophage infiltration. **F** Scatterplot showing correlation between SERPINF1 expression and activated dendritic cell infiltration

Quantitative CIBERSORT analysis of TCGA-BLCA samples revealed significant disparities in immune cell composition between high- and low-risk groups. High-risk patients exhibited elevated proportions of M0 and M2 macrophages and neutrophils, whereas low-risk patients were enriched for CD8⁺ T cells, activated NK cells, and plasma cells (Fig. 6A). Functional immune pathway analysis showed that high-risk tumors had significantly higher scores in APC co-inhibition, checkpoint, inflammation-promoting, and Type I IFN response pathways (Fig. 6B). Immune subtype classification based on gene expression divided 381 samples into four subtypes (C1–C4), with risk stratification correlating strongly with subtype distribution (*P* = 0.001). The immune subtypes C1–C4 correspond to Wound Healing (C1), IFN-gamma Dominant (C2), Inflammatory (C3), and Lymphocyte Depleted (C4), respectively. Low-risk cases were predominantly C1 (49%), while high-risk cases were mainly C2 (53%) (Fig. 6C). The C1 subtype resembles an immune state activated during wound healing, characterized by enhanced angiogenesis, high cellular proliferation, and extracellular matrix (ECM) remodeling. The C2 subtype is dominated by IFN-γ signaling within the immune microenvironment, representing a typical state of active anti-tumor immunity. Additionally, expression of DDR2 and SERPINF1 positively correlated with multiple immune cells and functions, most notably M2 macrophages (*p* < 0.05; Figs. 6D–F, S2). These findings are consistent with the hypothesis that the poor prognosis in high-risk patients may be driven by elevated TAM levels and impaired antigen presentation to T cells, as well as defective effector T-cell priming.

Comparing immune cell abundances, low-risk patients showed higher enrichment of memory B cells, plasma cells, CD8⁺ T cells, resting dendritic cells, and activated NK cells, indicating more robust antitumor surveillance. High-risk patients exhibited increased M0/M2 macrophages, neutrophils, and activated mast cells—hallmarks of an immunosuppressive or inflammatory microenvironment—along with elevated Tregs and monocytes, which may further weaken antitumor immunity (Fig. S3).

### TMB and TIDE analysis

**Fig. 7:**
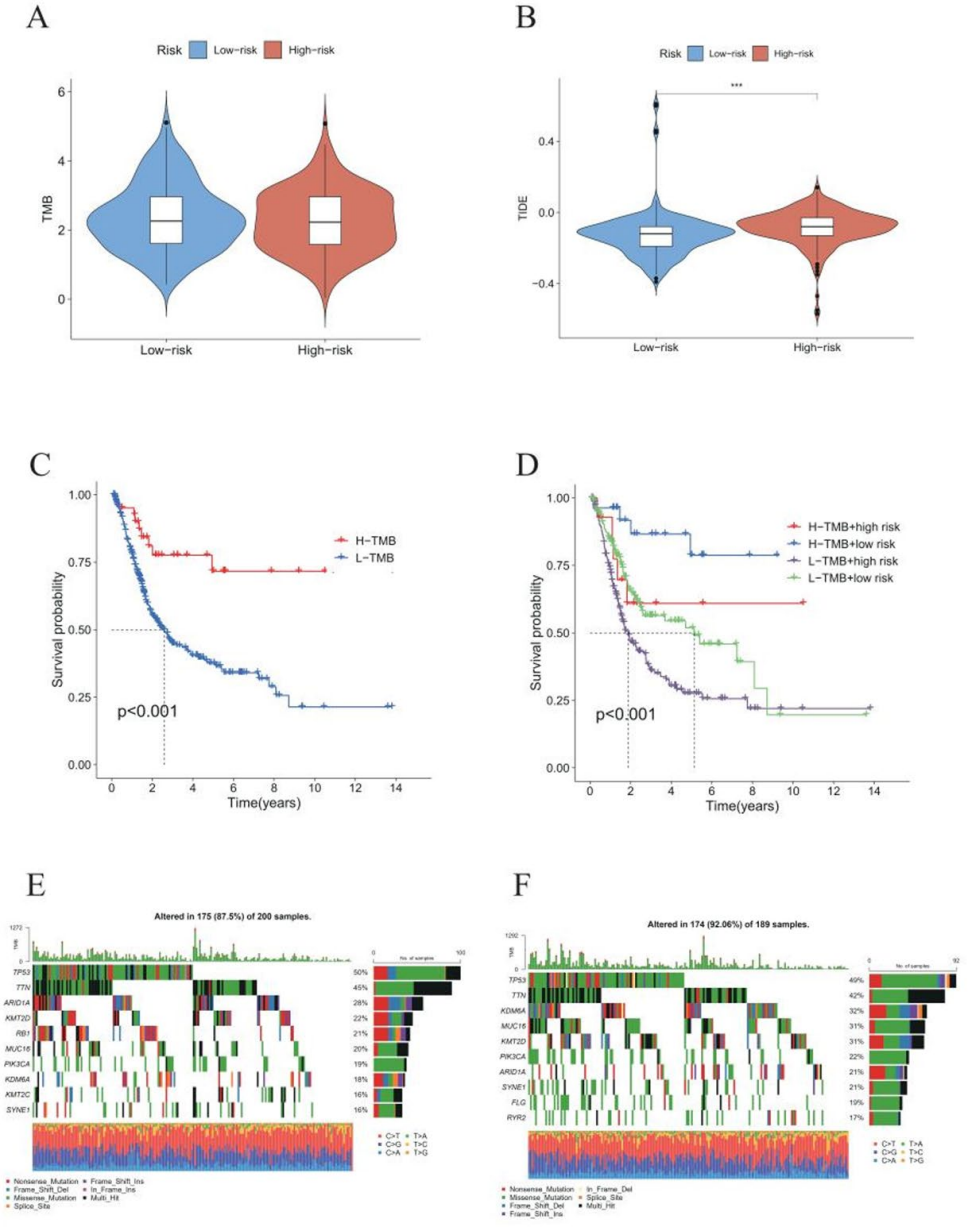
Analysis of tumor mutation burden (TMB), immune escape, mutation landscape, and prognostic evaluation in BLCA risk groups. **A** Comparison of TMB between high- and low-risk BLCA groups. **B** Differential TIDE immune escape scores between risk groups. **C** Survival analysis of TCGA-BLCA patients stratified by high and low TMB. The hazard ratio (HR) for high TMB versus low TMB was 2.15 (95% confidence interval [CI]: 1.62–2.86; log-rank *p* < 0.001). **D** Survival analysis based on combined stratification by TMB and risk scores. Using the “H-TMB + Low risk” group as the reference (HR = 1.00), the hazard ratios for other groups were as follows: “L-TMB + High risk”, HR = 3.21 (95% CI 2.15–4.78); “L-TMB + Low risk”, HR = 2.45 (95% CI 1.65–3.65); “H-TMB + High risk”, HR = 1.89 (95% CI 1.24–2.89). The overall difference among groups was significant (log-rank *p* < 0.001). **E**, **F** Mutation frequency comparison between high- and low-risk groups

**Fig. 8:**
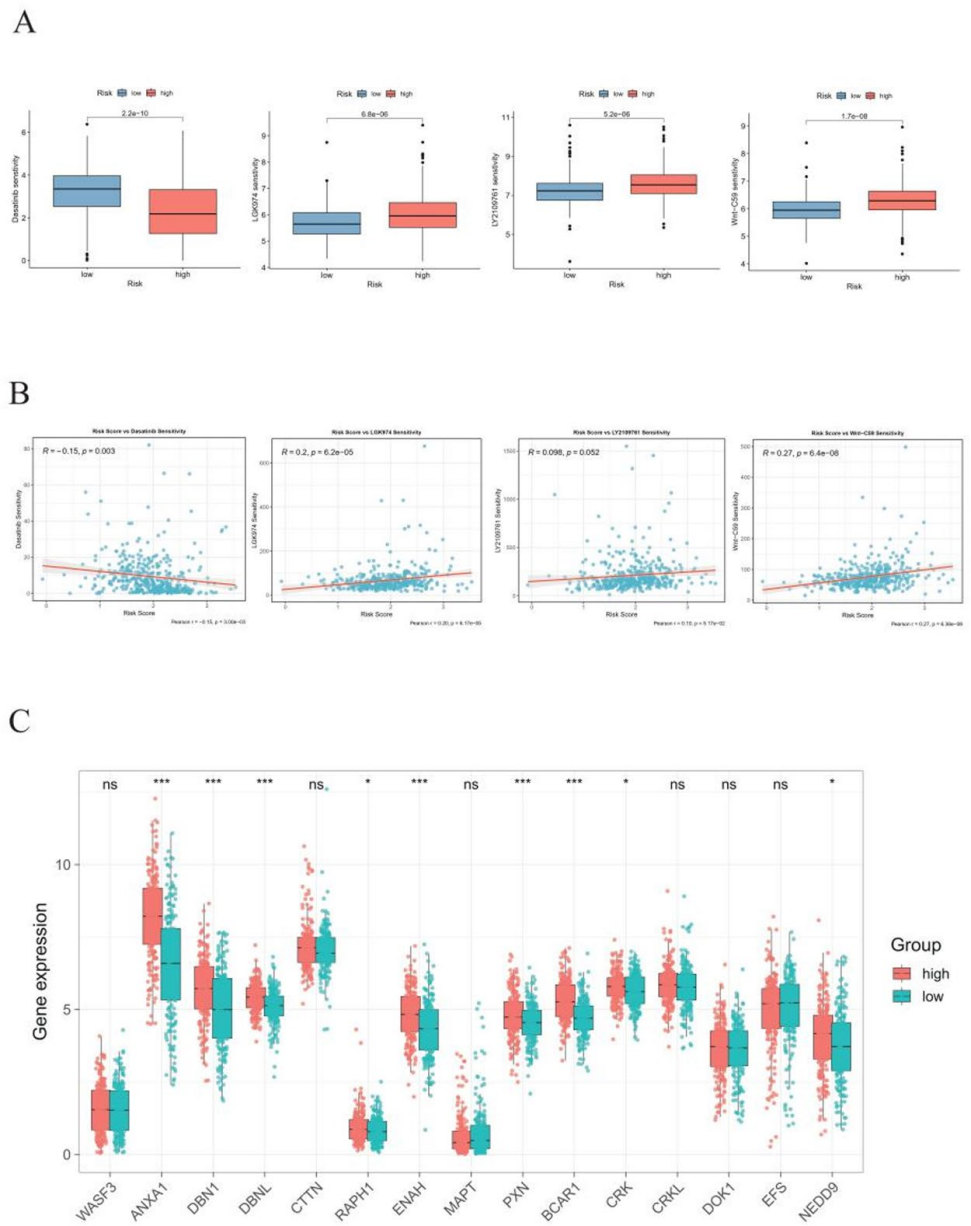
Targeted drug sensitivity and drug target gene expression analysis based on risk scores derived from metastasis- and basement membrane-related genes. **A** Comparison of small molecule inhibitor IC₅₀ values between high- and low-risk BLCA groups. **B** Correlation analysis between risk scores and drug sensitivity. **C** Differential expression analysis of DrugBank-identified drug target genes between risk groups

Given their links to immunotherapy response, we estimated tumor mutation burden (TMB) and TIDE scores for high- and low-risk groups. High-risk tumors exhibited slightly lower TMB than low-risk tumors (Fig. 7A) but had higher TIDE scores, indicating stronger immunosuppression and immune evasion (Fig. 7B). In the context of TCGA patients, cases exhibiting high-TMB demonstrated significantly superior overall survival outcomes in comparison to those exhibiting low-TMB (*P* < 0.001; Fig. 7C). Patients were stratified into four subgroups based on TMB and risk score: “high TMB and low risk,” “high TMB and high risk,” “low TMB and low risk,” and “low TMB and high risk.” Kaplan–Meier curves revealed that the “high TMB and low risk” group exhibited the most favourable outcomes, while the “low TMB and high risk” group demonstrated the least favourable outcomes. There were significant differences among all groups (*P* < 0.001; Fig. 7D). Furthermore, the mafTools R package was utilised to investigate the top 10 mutated genes in high- versus low-risk groups (*p* < 0.01). TP53 was identified as the most frequently mutated gene in both cohorts, with a marginally lower overall mutation rate observed in the high-risk cohort (Fig. 8C, D). While the overall mutation landscape was similar between risk groups, differences in mutation frequencies of driver genes such as RB1 and PIK3CA suggest subtle genomic instability variations across risk strata.

### Drug sensitivity analysis

To explore potential therapeutic vulnerabilities associated with the MBRG risk score, we applied the pRRophetic algorithm to evaluate the relationship between risk stratification and predicted IC₅₀ values of four small-molecule inhibitors (dasatinib, LGK974, LY2109761, and Wnt-C59) implicated in metastasis- and matrix remodeling–related pathways. Comparative analysis revealed that the predicted IC₅₀ of dasatinib was lower in the low-risk group, whereas predicted IC₅₀ values of LGK974, LY2109761, and Wnt-C59 were lower in the high-risk group (Fig. 8A).Consistently, the MBRG risk score showed a negative correlation with predicted dasatinib IC₅₀, and a positive correlation with predicted IC₅₀ values of LGK974 and Wnt-C59 (Fig. 8B). Moreover, several drug target genes retrieved from DrugBank exhibited significant differential expression between risk groups (*p* < 0.05; Fig. 8C), supporting the biological plausibility of the predicted drug response patterns. Collectively, these findings indicate that the MBRG risk score may serve as a computational framework for stratifying patients according to predicted drug sensitivity, rather than as a direct indicator of clinical drug efficacy. To further visualize the predicted drug sensitivity differences between risk groups, violin plots were generated to depict the distributions of predicted IC₅₀ values for the four representative agents in high- and low-risk patients (Fig. S4). Group differences were evaluated using the Wilcoxon rank-sum test, with FDR correction applied to account for multiple comparisons. In addition, effect sizes were quantified using Cliff’s delta to assess the magnitude and direction of the differences. Collectively, these analyses revealed distinct predicted drug response profiles associated with MBRG-based risk stratification.

### Single-cell and spatial transcriptomics of key gene distribution

**Fig. 9:**
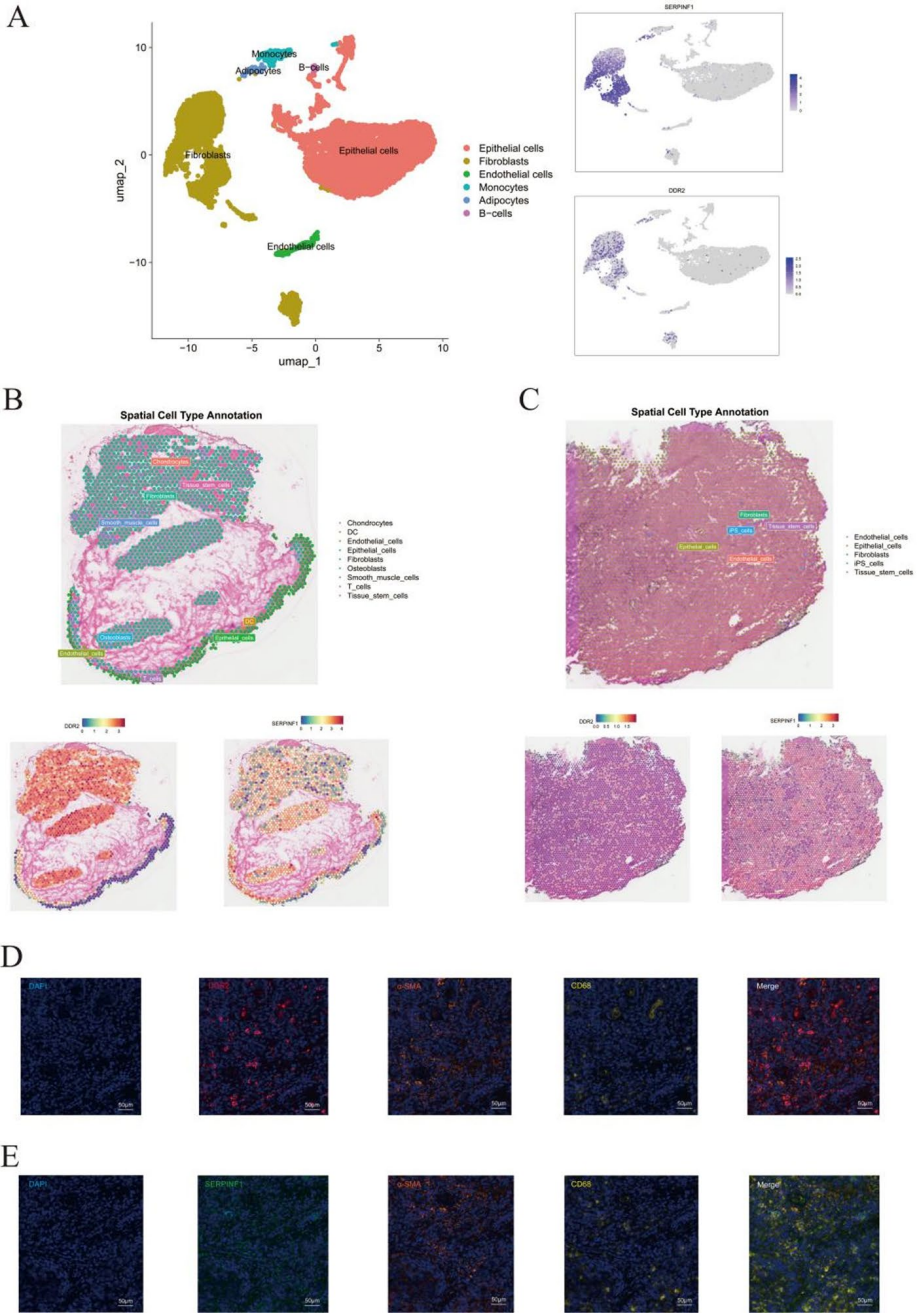
Cell lineage and spatial distribution of DDR2 and SERPINF1 in BLCA. **A** UMAP plot of single-cell transcriptomes colored by cell type, highlighting major populations including epithelial cells, fibroblasts, endothelial cells, monocytes, B cells, and adipocytes. Both DDR2 and SERPINF1 were predominantly expressed in fibroblasts. **B**, **C** Spatial transcriptomics analysis. Left: cell type annotation map of normal tissue; right: spatial expression heatmaps of DDR2 and SERPINF1 in tumor tissue. **D**, **E** Representative multiplex immunofluorescence (Opal-TSA) images acquired from the same section and the same field (ROI), showing DAPI nuclear staining and α-SMA and CD68 signals, with DDR2 (D) or SERPINF1 (E) channels displayed separately for clarity; merged images support the spatial transcriptomic findings. Scale bar, 50 μm

To delineate the cell-type specificity and spatial context of the EMT-associated prognostic genes DDR2 and SERPINF1, we integrated single-cell, spatial, and multiplex imaging analyses in BLCA. Analysis of the GSE129845 single-cell dataset revealed preferential expression of both DDR2 and SERPINF1 in fibroblasts rather than epithelial tumor cells (Fig. 9A). Spatial transcriptomics further localized DDR2 and SERPINF1 to fibroblast-enriched stromal niches adjacent to epithelial tumor nests across two independent specimens, with low expression observed in epithelial compartments (Fig. 9B–C). Consistently, multiplex immunofluorescence confirmed stromal localization: DDR2 and SERPINF1 were predominantly detected in SMA-α⁺ cancer-associated fibroblasts(CAFs) and were frequently juxtaposed with CD68⁺ myeloid cells, indicating a CAF–myeloid ecosystem at the tumor–stroma interface (Fig. 9D–E). Collectively, these findings reposition DDR2 and SERPINF1 as CAF-centric transcriptional programs within the BLCA TME. Notably, SERPINF1 was depleted in epithelial nests but retained in stromal CAFs, suggesting a stromal–epithelial gradient with potential implications for EMT, extracellular matrix remodeling, and tumor cell migration.

## Discussion

We developed a prognostic risk scoring model for BLCA based on genes associated with metastasis and basement membrane degradation (MBRGs), demonstrating its utility for predicting overall survival and immunotherapy response. Validation in independent TCGA and GEO cohorts confirmed consistent performance via nomogram calibration and ROC analyses. SHAP analysis quantified each gene’s impact on risk prediction, highlighting DDR2 as the most influential among the six-gene panel (DDR2, SERPINF1, SLIT2, HSPG2, ECM1, RECK). Integrated functional enrichment, immune deconvolution, and immunotherapy response analyses linked the MBRG risk score to invasion-related pathways, immune landscape remodeling, and therapeutic sensitivity. Drug sensitivity screening suggested tailored therapeutic options. Notably, single-cell RNA sequencing and spatial transcriptome sequencing analyses revealed higher expression of DDR2 and SERPINF1 in CAFs, implying that DDR2 may indirectly regulate EMT through CAF-tumor interactions.

Recent prognostic models for BLCA rely on diverse biological frameworks. Some emphasize immune checkpoint–related features while largely overlooking matrix remodeling, whereas others focus on CAF-related signatures without incorporating basement membrane degradation [36, 37]. In contrast, our model integrates classical BM-remodeling genes (ECM1, HSPG2, and RECK) with CAF-associated markers (DDR2 and SERPINF1), thereby capturing both physical barrier disruption and dynamic tumor–stroma interactions. This dual-layer design improves the detection of invasive potential and maintains robust predictive performance across independent TCGA and GEO cohorts. Within this framework, ECM1, HSPG2, and RECK constitute a coordinated basement membrane remodeling module [38–40].

From a clinical perspective, the MBRG signature appears to capture a permissive microenvironmental state that favors metastatic dissemination rather than reflecting isolated gene effects. The six genes included in the model collectively describe a sequence of events that lower the physical and biological barriers to tumor spread.

In high-risk patients, coordinated basement membrane destabilization, together with CAF-driven extracellular matrix remodeling, creates a microenvironment that facilitates tumor cell invasion, migration, and eventual dissemination. Importantly, this process may precede the development of clinically detectable metastases, which helps explain why patients classified as high risk by the MBRG score experience poorer survival outcomes even at comparable clinical stages.

Rather than acting as independent drivers, ECM1, HSPG2, and RECK primarily reflect structural vulnerability of the basement membrane, while DDR2 and SERPINF1 mark an activated stromal compartment that supports invasion through collagen remodeling and EMT-promoting signaling. Dysregulation of SLIT2 further removes constraints on cell motility. Together, these alterations define a metastasis-prone tumor microenvironment that is not fully captured by conventional staging parameters.

Clinically, this suggests that the MBRG risk score may help identify patients with biologically aggressive, invasion-prone tumors who could benefit from closer surveillance or early systemic intervention, even in the absence of overt metastatic disease at diagnosis.

The concurrent upregulation of ECM1 and HSPG2, together with downregulation of the MMP inhibitor RECK, reflects a shift toward BM destabilization, facilitating EMT-associated invasion and metastasis in high-risk BLCA patients.

Among the remaining prognostic genes, DDR2 and SLIT2 are closely linked to EMT regulation. Notably, single-cell and spatial transcriptomic analyses revealed that DDR2 expression is selectively enriched in cancer-associated fibroblasts, despite appearing reduced at the bulk tumor level. This apparent discrepancy underscores the cell type–specific and context-dependent role of DDR2 within the tumor microenvironment. CAF-derived DDR2 promotes ECM remodeling through collagen signaling and POSTN–integrin β1 activation, thereby creating a permissive stromal niche that indirectly facilitates EMT and tumor invasion [41]. Although this effect is masked in bulk transcriptomic analyses, it is captured by SHAP-based individual risk contributions, where DDR2 exerts a positive prognostic impact in specific patients. Together, these findings support DDR2 as a stromal-contextual risk factor whose prognostic relevance arises from CAF-driven microenvironmental remodeling rather than tumor cell–intrinsic expression. In parallel, aberrant SLIT2 expression in high-risk BLCA patients may compromise its migration-inhibitory function, further predisposing tumor cells to EMT and invasive behavior.

GSEA revealed that high-risk patients are enriched for gene sets involved in ECM–receptor interaction, cell adhesion, focal adhesion, and the PI3K–Akt signaling pathway—key drivers of tumor invasion, migration, and metastasis. ECM–receptor interactions and focal adhesions modulate adhesion strength and cytoskeletal dynamics to directly influence motility, while PI3K–Akt signaling governs cell survival, proliferation, and dissemination. The marked activation of these pathways in high-risk patients aligns with their poor prognosis, providing mechanistic support for our model’s biological rationale.

Immune profiling indicated pronounced immune escape in high-risk patients. Mechanistically, CAF-remodeled ECM upregulates CCL2 and CXCL12 to recruit M2 macrophages and regulatory T cells, establishing an immunosuppressive barrier [42, 43]. Zhang et al. showed CAF-derived CXCL12 drives PD-L1 accumulation on tumor cells, impairing CD8⁺ T cell cytotoxicity [44], while high CCL2 expression skews monocytes toward M2 polarization, which secrete IL-10 and TGF-β to inhibit effector T cell proliferation and activation [44]. In our cohort, elevated TIDE scores and CIBERSORT/ssGSEA quantification revealed a positive feedback loop between CCL2/CXCL12 upregulation and M2/MDSC & Treg enrichment, illustrating how ECM remodeling directly drives immune evasion.

By integrating age, grade, TNM stage, and the MBRG risk score, we developed a well-calibrated nomogram for individualized risk assessment in BLCA. Predictive drug sensitivity analysis suggested that high-risk patients exhibited lower predicted IC₅₀ values for the Wnt/β-catenin inhibitor LGK974 (1.2 µM vs. 3.8 µM; *p* < 0.001), which is consistent with the enhanced activation of β-catenin–related signaling pathways observed in this subgroup. Notably, early-phase clinical trials of WNT974 (LGK974) in solid tumors have demonstrated an acceptable safety profile and preliminary biological activity [45], supporting the translational relevance of Wnt pathway inhibition.Importantly, these findings should be interpreted as hypothesis-generating, as the pRRophetic-based predictions are derived from transcriptomic similarity to cancer cell line models rather than from direct clinical drug response data. Nevertheless, our results raise the possibility that risk-adapted therapeutic strategies, such as prioritizing Wnt pathway–targeted agents in high-risk patients and avoiding overtreatment in low-risk patients, may warrant further evaluation in prospective clinical studies.

This study integrates BM remodeling and metastasis gene signatures with tumor microenvironment and immune infiltration analyses, providing novel prognostic insights and mechanistic depth for BLCA. Several limitations of this study should be acknowledged. First, the number of samples included in the single-cell RNA sequencing analysis was relatively small (*n* = 3), which limits our ability to comprehensively characterize the full heterogeneity of CAF subpopulations in BLCA. As a result, we did not attempt to define distinct CAF functional states (such as myCAF or iCAF) or draw conclusions regarding their relative prevalence across patients.

Importantly, our single-cell analyses were not used as the primary basis for prognostic modeling, but rather to provide cell type–specific context for key genes identified from large-scale bulk transcriptomic cohorts. The CAF-enriched expression of DDR2 and SERPINF1 was consistently supported by spatial transcriptomics, tissue microarray validation, and reproducible prognostic effects across TCGA and multiple independent GEO datasets. These complementary approaches help mitigate the limited generalizability inherent to small single-cell sample sizes.

Nevertheless, larger single-cell and spatial profiling studies will be necessary to fully delineate CAF heterogeneity in BLCA and to validate whether DDR2⁺ CAFs represent a conserved functional subtype across diverse clinical settings. Future work integrating high-throughput spatial transcriptomics with longitudinal clinical cohorts will be essential to refine stromal-targeted therapeutic strategies.

## Conclusion

Our MBRG-based prognostic model for BLCA showed promising predictive performance in our retrospective cohorts, elucidated the synergistic interplay of basement membrane remodeling, EMT, and immune suppression, and, through SHAP and single-cell analyses, confirmed the pivotal roles of DDR2 and SERPINF1 in CAFs, thereby potentially providing a novel tool for personalized prognostic assessment and guiding precision therapy strategies.

## Supplementary Information

Below is the link to the electronic supplementary material.

Supplementary Material 1.

Supplementary Material 2.

## Data Availability

The datasets supporting the findings of this study are publicly available in the following repositories: The Cancer Genome Atlas (TCGA): All TCGA data analyzed in this manuscript can be accessed via the Genomic Data Commons (GDC) Portal at https://portal.gdc.cancer.gov/. Specific datasets were retrieved using the TCGA project identifiers through the GDC Data Portal interface or API. Gene Expression Omnibus (GEO): Processed and raw RNA-seq/microarray data were obtained from the GEO database at the National Center for Biotechnology Information (NCBI) https://www.ncbi.nlm.nih.gov/geo/. The data from GEO can be found under accession numbers GSE32894, GSE129845 and GSE285715. Further inquiries can be directed to the corresponding author.

## Abbreviations

SHAP: SHapley Additive exPlanations
MBRGs: Metastasis–basement membrane–related genes
NMIBC: Non-muscle invasive
MIBC: Muscle-invasive bladder cancer
TAMs: Tumor-associated macrophages
ICIs: Immune checkpoint inhibitors
BLCA: Bladder cancer
TCGA: The Cancer Genome Atlas
BM: Basement membrane
ROC: Receiver operating characteristic
AUC: Area under the curve
CNV: Copy number variation
EMT: Epithelial-Mesenchymal Transition
TMB: Tumor mutation burden
GSEA: Gene Set Enrichment Analysis
