# Supplementary material for: Single-cell and immune-context integration identifies basement-membrane/metastasis signatures that sharpen bladder-cancer diagnosis and prognosis
Source: Discov Oncol. 2026 Jan 14;17:278. doi: 10.1007/s12672-026-04440-3 (PMC12891303; doi:10.1007/s12672-026-04440-3)
Supplement: Supplementary file 1 — Supplementary Material 1. [file 12672_2026_4440_MOESM1_ESM.docx]

**Supplementary Figure S1. Prognostic relevance of individual metastasis- and matrix-related genes in BLCA**

**
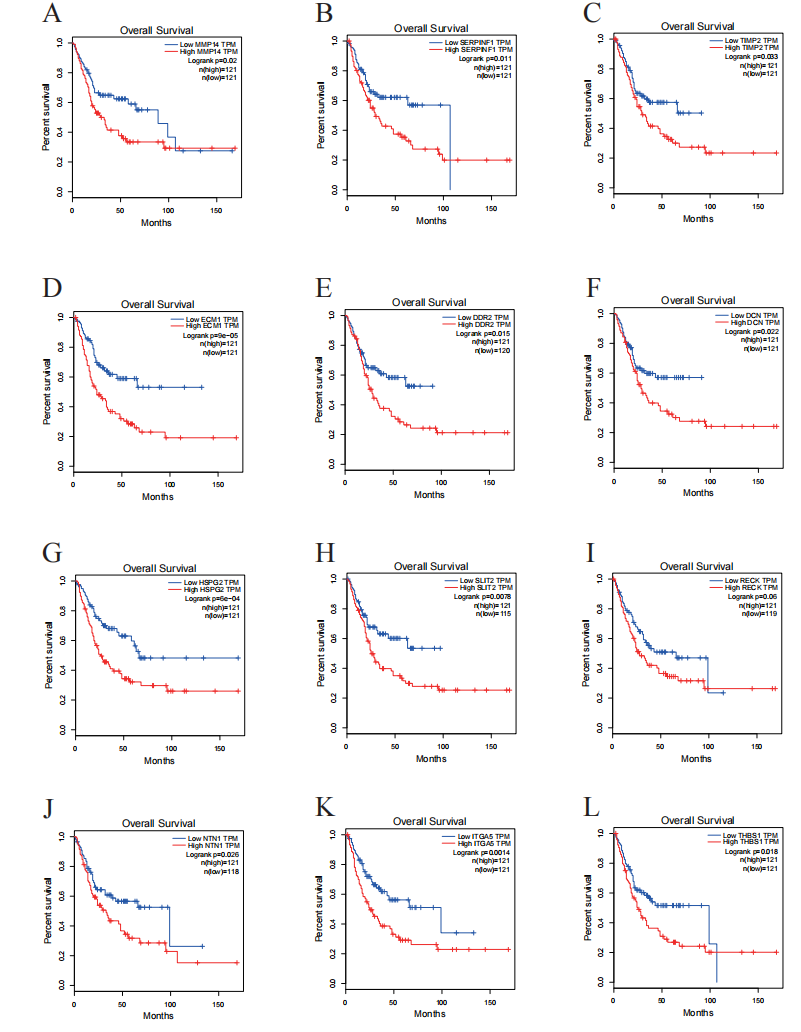
**

**Figure S1. Kaplan–Meier survival analysis of individual metastasis- and basement membrane–related genes in BLCA.**

Kaplan–Meier curves showing overall survival differences between patients with high and low expression levels (TPM-based median split) of selected metastasis- and extracellular matrix–associated genes, including MMP14 (A), SERPINF1 (B), TIMP2 (C), ECM1 (D), DDR2 (E), DCN (F), HSPG2 (G), SLIT2 (H), RECK (I), NTN1 (J), ITGA5 (K), and THBS1 (L). Survival differences were assessed using the log-rank test. These results demonstrate that multiple components involved in extracellular matrix remodeling, stromal activation, and cell migration are individually associated with patient prognosis, supporting their integration into the MBRG prognostic signature.

**Supplementary Figure S2. Association between DDR2 expression and immune cell infiltration**

**
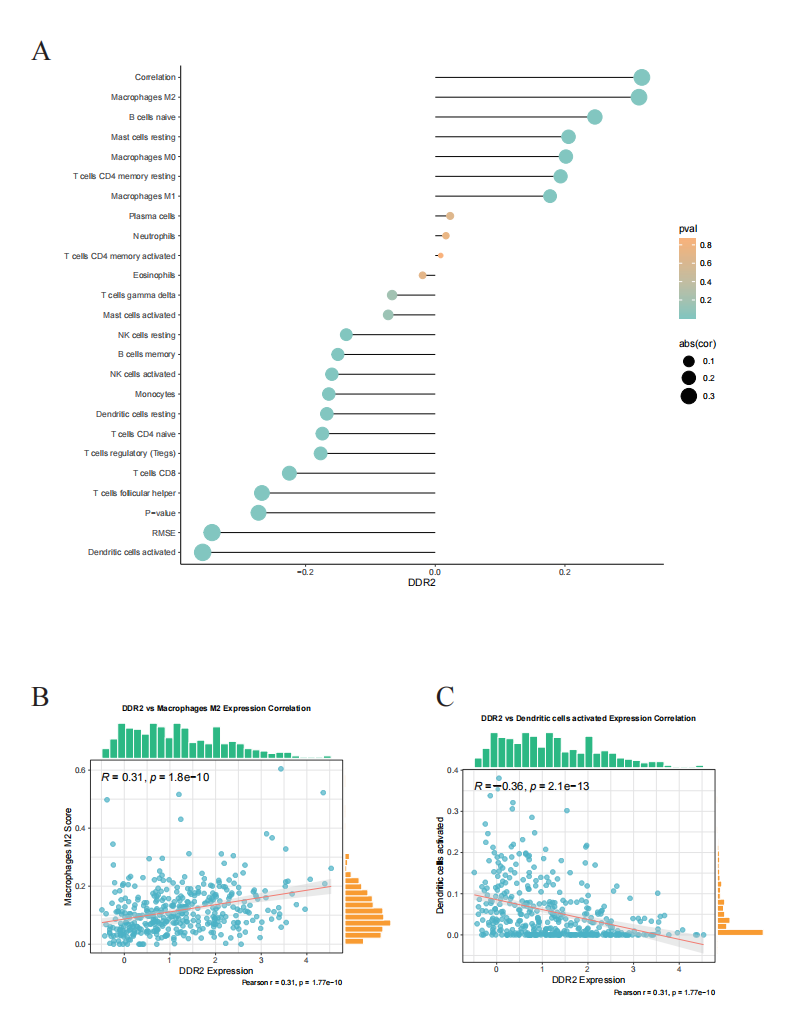
**

**Figure S2. Correlation between DDR2 expression and immune cell infiltration patterns in BLCA.**

(A) Bubble plot summarizing the correlations between DDR2 expression and the inferred abundance of multiple immune cell populations estimated using immune deconvolution analysis. Dot size represents the absolute value of the correlation coefficient, and color indicates statistical significance.

(B–C) Scatter plots illustrating the positive correlation between DDR2 expression and macrophage M2 scores (B), and the inverse association between DDR2 expression and activated dendritic cell infiltration (C). Pearson correlation coefficients and corresponding P values are shown. These findings suggest that elevated DDR2 expression is associated with an immunosuppressive tumor microenvironment, characterized by increased M2 macrophage polarization and reduced antigen-presenting cell activity.

**Supplementary Figure S3. Immune landscape differences between MBRG-defined risk groups**

**
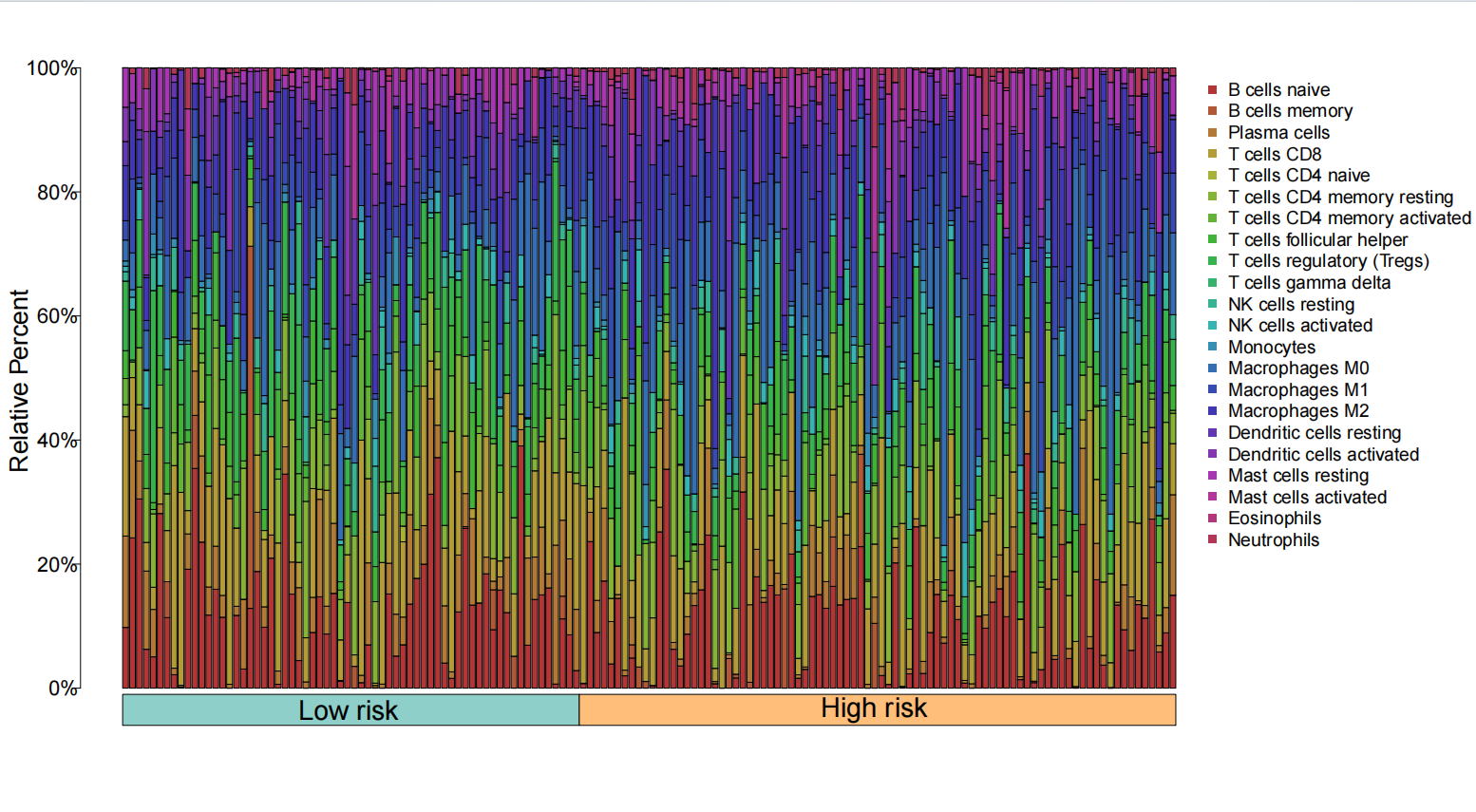
**

**Figure S3. Immune cell composition in low- and high-risk BLCA patients stratified by the MBRG risk score.**

Stacked bar plots showing the relative proportions of 22 immune cell types estimated by CIBERSORT in individual BLCA samples, grouped according to low- and high-risk categories defined by the MBRG prognostic signature. Differences in immune composition highlight enrichment of immunosuppressive cell populations, including M2 macrophages and regulatory T cells, in the high-risk group, whereas low-risk tumors exhibit a comparatively more immune-active profile. These patterns further support a link between MBRG-defined risk status, stromal remodeling, and immune escape.

**Supplementary Figure S4. Predicted drug sensitivity differences between high- and low-risk BLCA patients.**

**
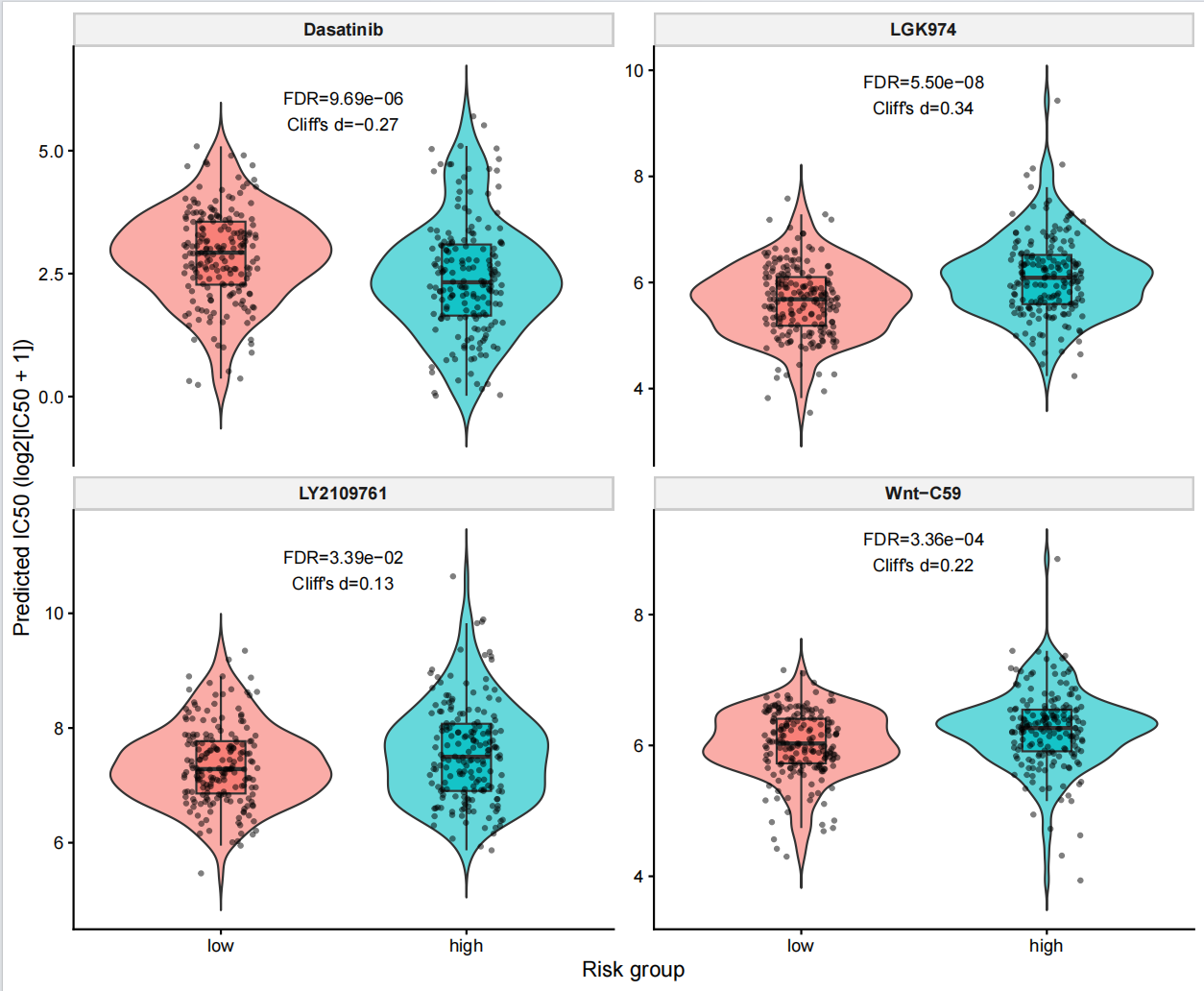
**

**Figure S4. Predicted drug sensitivity differences between high- and low-risk BLCA patients.**

Violin plots illustrate the distribution of predicted IC₅₀ values (log2[IC₅₀ + 1]) for four small-molecule inhibitors (Dasatinib, LGK974, LY2109761, and Wnt-C59) in the low- and high-risk groups defined by the MBRG risk score. Individual dots represent individual patients, and embedded boxplots indicate the median and interquartile range. Statistical significance between groups was assessed using the Wilcoxon rank-sum test, with false discovery rate (FDR) correction applied for multiple comparisons. Effect sizes were quantified using Cliff’s delta (d). Low-risk patients exhibited lower predicted IC₅₀ values for Dasatinib, whereas high-risk patients showed increased predicted sensitivity to LGK974, LY2109761, and Wnt-C59, suggesting distinct drug response patterns associated with metastatic risk stratification.
